# Isolated Pulmonary Embolism following Shoulder Arthroscopy

**DOI:** 10.1155/2014/279082

**Published:** 2014-12-07

**Authors:** Nicole H. Goldhaber, Christopher S. Lee

**Affiliations:** Stetson Powell Orthopaedics and Sports Medicine, 191 S. Buena Vista Street, Suite 470, Burbank, CA 91505, USA

## Abstract

Pulmonary embolism (PE) following shoulder arthroscopy is a rare complication. We present a unique case report of a 43-year-old right-hand dominant female who developed a PE 41 days postoperatively with no associated upper or lower extremity DVT. The patient had minimal preoperative and intraoperative risk factors. Additionally, she had no thromboembolic symptoms postoperatively until 41 days following surgery when she developed sudden right-hand swelling, labored breathing, and abdominal pain. A stat pulmonary computed tomography (CT) angiogram of the chest revealed an acute PE in the right lower lobe, and subsequent extremity ultrasounds showed no upper or lower extremity deep vein thrombosis. After a thorough review of the literature, we present the first documented isolated PE following shoulder arthroscopy. Although rare, sudden development of an isolated PE is possible, and symptoms such as sudden hand swelling, trouble breathing, and systemic symptoms should be evaluated aggressively with a pulmonary CT angiogram given the fact that an extremity ultrasound may be negative for deep vein thrombosis.

## 1. Introduction

Pulmonary embolism (PE) following shoulder arthroscopy is an extremely rare but potentially fatal complication. In the general population, PE is a clinical risk with a reported incidence of 23 per 100,000 patients per year [1]. It is responsible for 200,000 deaths each year in the United States alone [1]. In a systematic review of all literature published prior to March 31, 2012, Dattani et al. reported that the incidence of VTE following arthroscopic surgery of the shoulder was 0.038% from 92,440 procedures [2]. Of these, only 1–4% of all DVTs involved the upper limbs [3]. The incidence of pulmonary embolism following shoulder surgery is even lower, with only a handful of case reports published (Table 1). In each case report, the patient had an associated deep vein thrombosis that led to the pulmonary embolism most of which occurred within three weeks of surgery. We present a case report of a patient who developed a pulmonary embolism nearly six weeks following a shoulder arthroscopy with no associated deep vein thrombosis in an extremity and minimal preoperative and operative risk factors.

## 2. Case Presentation

GK is a 43-year-old right-hand dominant nonsmoking female who presented to our office with right shoulder pain. The patient had symptoms for nine months following a motor-vehicle accident where she was a restrained passenger. The patient stated she had severe pain at the anterolateral aspect of her right shoulder, difficulty lifting greater than five pounds, limited overhead motion, and subjective catching. Physical examination showed her height, weight, and body mass index were 61 inches, 135 pounds, and 25.51, respectively. After history, physical, and radiographic examination, the patient was diagnosed with a rotator cuff strain, long head biceps tenosynovitis, and acromioclavicular joint osteolysis. Over the course of one year, the patient attended 36 sessions of physical therapy, participated in a home exercise program and underwent a subacromial cortisone injection with only temporary relief. Due to her lack of progress with conservative measures, an MRI arthrogram was obtained which showed a moderate bursal-sided tear of the supraspinatus, moderate acromioclavicular joint osteolysis, and a type II superior labrum anterior posterior (SLAP) lesion. A discussion of nonoperative versus operative treatment options, outcomes, and complications occurred and the patient elected to go forward with surgery.

After being brought into the operating room, the patient underwent an interscalene nerve block for intraoperative and postoperative pain control using a nerve stimulator. Bilateral lower extremity sequential compression devices were applied for deep vein thrombosis prophylaxis. General endotracheal tube anesthesia (GETA) was administered. The patient was then positioned into the modified lateral decubitus position (patient rolled back 30 degrees) using a foam wedge with all bony prominences well padded. An axillary roll was placed one hand breadth distal to the axilla, and the patient was secured with Velcro straps. After sterile draping and time-out, the patient's operative extremity was placed in ten pounds of balanced suspension in the scapular plane.

Upon shoulder arthroscopy, the patient was found to have a type II SLAP lesion with a long head biceps that had inflammation and fraying. The articular side of the rotator cuff had mild fraying. When viewing the subacromial space, the patient's rotator cuff was found to have significant fraying, fragmentation, and thinning of the bursal side (type A1, B3). When probed, the supraspinatus was found to be of inadequate quality due to intrasubstance tearing. In addition, the acromioclavicular joint was arthritic. As a result, the patient underwent an arthroscopic take-down and repair of the rotator cuff, a distal clavicle excision, and a miniopen subpectoralis long head biceps tenodesis. The duration of the surgery was one hour and 45 minutes with the duration of balanced suspension one hour and 20 minutes. There were no complications intraoperatively. The patient was placed into a sling with an abduction pillow and awoken from the anesthesia with no incident. She was taken to the postanesthesia care unit in good condition.

At her initial postoperative visit six days after surgery, the patient presented with occasional pain in her shoulder with moderate swelling. She denied nausea, vomiting, fever, chills, and sweats and had no calf tenderness. She was not menopausal. She was taking Percocet as needed as with appropriate relief but otherwise denied any additional medication history. Her incisions were clean and dry, and the stitches were removed and steri-strips applied. The patient was instructed to continue wearing her sling and abduction pillow except when performing twice daily Codman's exercises. Exercises for elbow, wrist, and finger range of motion were demonstrated, and the patient was advised to perform these twice per day. 

At four weeks postoperatively, the patient's pain was well-controlled with no requirement for pain medication, and her wounds were well-healed. She denied fevers, chills, and calf pain. Her sling was discontinued and formal physical therapy was initiated as per our standard rotator cuff repair and biceps tenodesis protocol.

**Figure 1:**
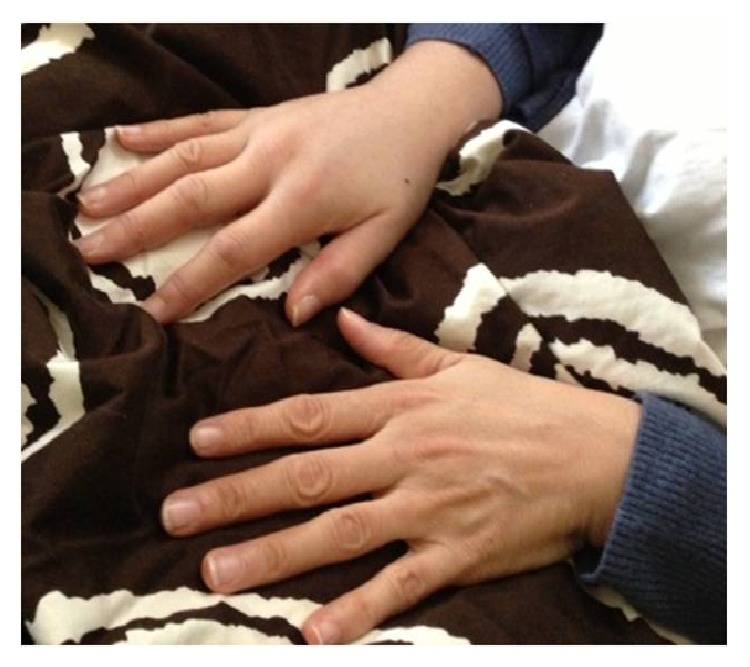
Photograph sent by GK to our office depicting her sudden right-hand swelling.

41 days postoperatively, the patient called our office with new-onset right-hand swelling and trouble breathing. She also reported that she had been feeling “flu-like symptoms” and stomach pains. She had no calf pain or lower extremity swelling. The patient was able to send our office a picture of her swollen hand over a secure email service with patient identification removed (Figure 1). After seeing the photograph, she was advised to immediately follow up with us where upper extremity swelling and slightly labored breathing were confirmed.

**Figure 2:**
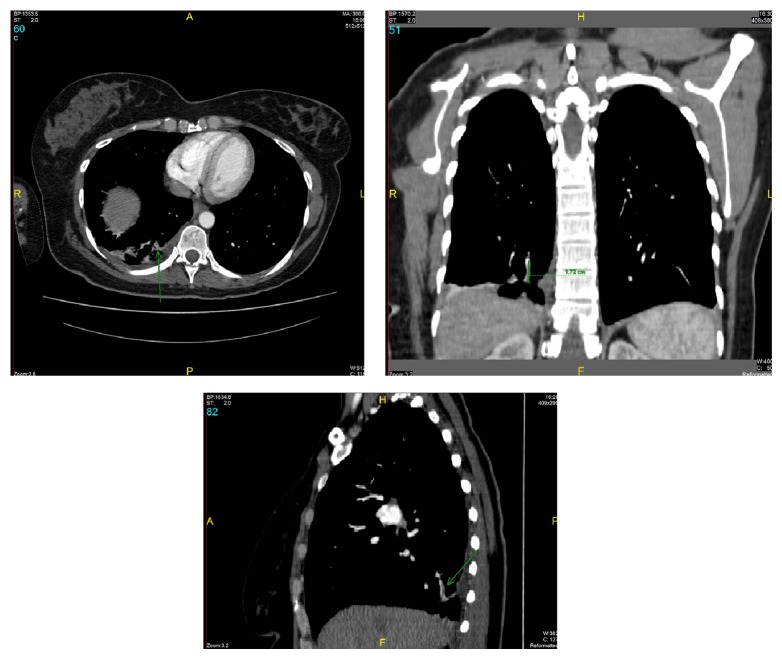
Computed tomography angiogram (CTA) transverse, coronal, and sagittal images depicting the presence of an acute pulmonary embolism (PE) in the subsegmental posterior basal segment artery of the right lower lobe indicated by the green arrows.

Vital signs were stable and we ordered a stat computed tomography (CT) pulmonary angiogram, which revealed that the patient had an acute pulmonary embolism (PE) in the subsegmental posterior basal segment artery of the right lower lobe (Figure 2). A stat hematology consult was arranged, and the patient was admitted through the emergency room to the hematology service for further evaluation and management. The patient was started on a therapeutic dose of Lovenox and subsequently transitioned to per-oral Xarelto. Three days after admission, she underwent a bilateral upper extremity venous duplex ultrasound that was negative for deep vein thrombosis (DVT). An immediate ultrasound upon admission was not performed due to the hematologist service's need to stabilize the patient prior to additional testing. She had no pain, swelling, or tenderness in the left upper extremity or bilateral lower extremities. A full hematology analysis was performed and was negative for any predisposing genetic factors for VTE. In assessing the patient's objective risk factors for VTE, a Revised Geneva Score was calculated and resulted in a score of two, corresponding to low risk for DVT [15].

Three days after initiation of anticoagulation therapy, the patient's constitutional symptoms and discomfort with breathing resolved. Two weeks later, her upper extremity swelling resolved. Although her preoperative pain was completely resolved, the patient developed postoperative stiffness, and at six months postoperatively she had a protracted humeral head with active forward elevation 130 degrees, external rotation 45 degrees, and internal rotation to L5. Her strength with regard to her supraspinatus, infraspinatus, subscapularis, and biceps was 5/5 with no pain, and a postoperative MRI at 6 months postoperatively showed complete healing of the supraspinatus repair and long head biceps tenodesis.

## 3. Discussion

Pulmonary emboli that develop after upper extremity surgery arise mainly from the ipsilateral axillary subclavian venous system or either of the lower extremities [16]. General risk factors for DVT following shoulder surgeries documented in the literature include the lateral decubitus position with suspension of the limb, prolonged surgical time, use of an interscalene nerve block, increasing age, personal or family history of thromboembolism, smoking, diabetes mellitus, and obesity [7, 9]. Surgical risk factors include venous irritation or compression by a mechanical shaver, subcutaneous edema around the shoulder from fluid extravasation, and inadequate positioning of the arm [5]. Medical risk factors for VTE in upper and lower extremities include varicosities, smoking, obesity, diabetes mellitus, rheumatoid arthritis, ischemic heart disease, neoplasia, and venous stasis [2, 7, 9]. Our standard VTE prophylaxis for shoulder arthroscopy is to apply compression stockings and sequential compression devices intraoperatively. We do not use mechanical or chemical prophylaxis methods unless the patient is over 65 years old, is a tobacco user, has a body mass index greater than 30, takes oral contraceptives and/or hormone replacement therapy, or has a previous history and/or family history of DVT. We present a case of a pulmonary embolism following shoulder arthroscopy with minimal preoperative or medical risk factors and minimal surgical risk factors with no concomitant extremity DVT. Although rare, an isolated pulmonary embolism can certainly occur following upper extremity surgery, and given the severity of the complication, we feel it is important to be able to recognize upper extremity swelling, difficulty in breathing, and constitutional symptoms as signs of a potentially adverse event. We feel that should these symptoms occur following an upper extremity procedure, CT pulmonary angiogram should be the test of choice to diagnose pulmonary embolism.

Our case also suggests that there is no clear time period beyond which a patient is no longer at risk of a pulmonary embolism. While previously reported pulmonary emboli following shoulder arthroscopy occur typically no later than 29 days postoperatively, our case presented nearly six weeks postoperatively [7]. This was long after discontinuation of upper extremity immobilization and well into the postoperative rehabilitation course.

Burkhart and colleagues were the first to report a case of thromboembolism which developed in a 32-year-old man three days after shoulder arthroscopy in 1990 [4]. Since that time, we have found only five reports of PE following shoulder arthroscopy, all of which occurred within 29 days of surgery [5, 7–9, 11]. Willis et al. reported that the risk of PE in the shoulder arthroplasty literature ranges from 0.2% to 2% with a mortality rate of 1% [16]. It is likely that these percentages are slightly higher than observed for other procedures due to the higher average age of patients which undergo shoulder replacement surgery. With regard to shoulder procedures in general, Jameson et al. reported the PE incidence from 0.01% to 0.52% following 80,227 shoulder procedures [17]. Dattani et al. reported that the incidence of VTE following arthroscopic surgery of the shoulder was 0.038% from 92,440 procedures [2]. In each of these studies, the range of symptoms varied from asymptomatic to pain and/or swelling of the arm, a prominence of superficial veins in the upper arm and chest, cyanosis of the hand, numbness and tingling of the fingers, and functional impairment.

Given the wide variety of presentations, we feel that PE cannot be reliably diagnosed on the basis of history and examination alone. While ultrasound with venous imaging is portable, relatively inexpensive and has few contraindications, it is ideally suited for the diagnosis of a DVT and not PE. It is also operator dependent and less accurate for certain DVT formations and locations, and false negative studies may occur in the presence of a calf DVT, proximal DVT in asymptomatic patients or in the presence of a thrombosed duplicated venous segment [18]. In our case, an upper extremity ultrasound would have been negative and could possibly have led to missing the PE. On the other hand, spiral CT pulmonary angiogram has been associated with greater than 95% sensitivity and specificity and has the greatest sensitivity for emboli in the main, lobar, or segmental pulmonary arteries due to its ability to define nonvascular structures such as lymphadenopathy, lung tumors, emphysema, and other parenchymal abnormalities [18]. Ultrasonography has been reported to have a negative predictive value of 97% to 98%, whereas computed tomography (CT) scans detect all clinically relevant PE and a large number of alternative diagnoses [19]. As a result, if a patient presents with upper extremity swelling, constitutional symptoms, and discomfort with breathing following shoulder surgery, we would recommend first obtaining a spiral CT pulmonary angiogram to evaluate for PE. If the spiral CT pulmonary angiogram is negative, then we would recommend obtaining an upper extremity ultrasound to evaluate for DVT.

Kim et al. reported a fatal PE caused by thrombosis of the contralateral axillary vein after arthroscopic right rotator cuff repair [10]. If precautions are not taken and symptomatic patients are not screened appropriately, the consequences are dire. Given the extreme rarity of the condition, we feel that the cost of first evaluating upper extremity swelling with constitutional symptoms and discomfort with breathing with spiral CT pulmonary angiogram is well worth the risk of fatality. Although developing a PE following elective arthroscopic shoulder surgery is extremely rare, surgeons should be aware of its possibility and be able to recognize sentinel symptoms. As seen with our patient, we believe that a pulmonary embolism can occur in the absence of an extremity DVT. Thorough and aggressive evaluation with spiral CT pulmonary embolism should be the first-line test when a patient presents with upper extremity swelling, constitutional symptoms, and discomfort with breathing following shoulder arthroscopy.

## Figures and Tables

**Table 1:** Previously published case reports of pulmonary embolism following shoulder arthroscopy.

Authors	Age	Days after surgery	Male/female
Burkhart 1990 [4]	32	3	Male
Cortés et al. 2007 [5]	43	7	Male
Creighton and Cole 2007 [6]	52	10	Female
Edgar et al. 2012 [7]	26	14	Male
Edgar et al. 2012 [7]	45	29	Female
Edgar et al. 2012 [7]	59	2	Male
Garofalo et al. 2010 [8]	21	21	—
Hariri et al. 2009 [9]	25	10	Male
Hariri et al. 2009 [9]	63	30	Male
Hariri et al. 2009 [9]	63	7	Male
Kim et al. 2010 [10]	45	1	Female
Kuremsky et al. 2011 [11]	18	28	Male
Kuremsky et al. 2011 [11]	45	7	Male
Kuremsky et al. 2011 [11]	48	42	Male
Kuremsky et al. 2011 [11]	50	21	Male
Polzhofer et al. 2003 [12]	48	7	Male
Randelli et al. 2010 [13]	—	10	—
Yamamoto et al. 2013 [14]	72	6	Female
